# Solving the Single-Vehicle Self-Driving Car Trolley Problem Using Risk Theory and Vehicle Dynamics

**DOI:** 10.1007/s11948-019-00102-6

**Published:** 2019-04-01

**Authors:** Rebecca Davnall

**Affiliations:** Department of Philosophy, University of Liverpool, Brownlow Hill, Liverpool, L69 7ZX UK

**Keywords:** Self-driving cars, The trolley problem, Automation, Ethics, Risk, Vehicle dynamics

## Abstract

Questions of what a self-driving car ought to do if it encounters a situation analogous to the ‘trolley problem’ have dominated recent discussion of the ethics of self-driving cars. This paper argues that this interest is misplaced. If a trolley-style dilemma situation actually occurs, given the limits on what information will be available to the car, the dynamics of braking and tyre traction determine that, irrespective of outcome, it is always least risky for the car to brake in a straight line rather than swerve.

## A Generic Self-Driving Car Dilemma

Recent high-profile advances in self-driving car technology have prompted a wave of interest in the ethical implications of the design and deployment of these systems. This attention has coalesced around a set of ethical dilemmas which, especially in the popular imagination,^1^ are held to be broadly analogous to the trolley problem (Foot 1967). In some cases this framing has been used to suggest that the development of road-ready self-driving car software will require ‘solving’ the trolley problem itself.

The dilemmaic framing of these cases is a mistake born of failure to appreciate important and well-established empirical data from the field of vehicle dynamics. In effect, all such hypotheticals *as they apply to self*-*driving cars in the real world* will be optimally resolved if the car in question performs its best emergency stop procedure. The car will not actually face the kind of dilemma envisaged, because no action available to the car, given the information it is reasonable to anticipate will be available to it, will be clearly morally preferable or equal to maximally-efficient braking in a straight line.

This solution, set out in detail in the third section of this paper, is sound for cases which involve only a single vehicle; additional vehicles, regardless of whether they are self-driving or not, introduce complexities which are briefly addressed in the final section. This is an important limitation of the solution. In another sense, however, this solution is quite general: it will be established below—again on practical grounds—that very little information about the specific scenario *apart* from the number of vehicles involved is relevant to the question of what the vehicle ought to do.

To that end, it will be useful to establish a minimally-detailed version of the hypothetical. Most of the clear examples in the literature are quite vivid, featuring details which must be examined later; at this point a representative sample of scenarios are presented only to draw out their common elements.

A commonly-cited and clear example is Jan Gogoll and Julian F. Müller’s *tunnel case*:

Imagine you are sitting in your autonomous car going at a steady pace entering a tunnel. In front of you is a school bus with children on board going at the same pace as you are. In the left lane there is a single car with two passengers overtaking you. For some reason the bus in front of you brakes and your car cannot brake to avoid crashing into the bus. There are three different strategies your car can follow: First, brake and crash into the bus, which will result in the loss of lives on the bus. Second, steer into the passing car on your left—pushing it into the wall, saving your life but killing the other car’s two passengers. Third, it can steer itself (and you) into the right hand sidewall of the tunnel, sacrificing you but sparing all other participants’ lives. (2017: 683)

Patrick Lin offers the following version:

Imagine in some distant future, your autonomous car encounters this terrible choice: it must either swerve left and strike an 8-year old girl, or swerve right and strike an 80-year old grandmother. Given the car’s velocity, either victim would surely be killed on impact. If you do not swerve, both victims will be struck and killed; so there is good reason to think that you ought to swerve one way or another. (2016: 69–70)

In a Stapledon lecture at the University of Liverpool, Fiona Woollard offered two examples:

Swerve: An autonomous car with two passengers (mother and child) is driving at the speed limit on a 40 mph road when three drunken pedestrians stumble into the road. The only way to avoid hitting the pedestrians is to swerve the car into a wall, risking the life of the mother and child.

Tree: A tree suddenly falls into the road in front of a driverless car carrying 5 passengers. The only way to avoid hitting the tree is to swerve onto one of the pavements. A single pedestrian is walking on the right hand pavement. A crowd of school children is waiting for a bus on the left hand pavement. (2017)

And Sven Nyhom and Jilles Smids offer the *truck case*:

A self-driving car with five passengers approaches a conventional car (e.g. a heavy truck) that for some reason suddenly departs from its lane and heads directly towards the self-driving car. In a split-second, the self-driving car senses the trajectory and the likely weight of the oncoming truck. It calculates that a high-impact collision is inevitable, which would kill the five passengers, unless the car swerves towards the pavement on its right-hand side. There, unfortunately, an elderly pedestrian happens to be walking, and he will die as a result if the self-driving car swerves to the right and hits him. This is the sort of situation in which the human passengers of a self-driving car cannot take control quickly enough. (2016: 1278)

These examples can all be described as variants of the trolley problem because they all involve a decision between clearly-delineated options, each of which at least implicitly leads to certain harm (or in these cases, certain collisions), and where the chief difficulty arises from a lack of general consensus about which harms would be worse. Cases of this kind are supposed to raise questions of which option to take and which harm thereby to cause.

The number of options said to be available to the car differs among the five cases. In the tunnel case, Lin’s case and Woollard’s tree case, there are three options—to continue straight, to swerve left or to swerve right. In Woollard’s swerve case and the truck case, there are only two: swerve, or continue straight on. It can be safely assumed in the latter two cases that the swerve is in the direction away from the oncoming traffic lane. A car that veers into the oncoming traffic lane risks collisions at much higher relative speeds since its speed will be added to the speed of oncoming vehicles; for similar reasons, even where the oncoming traffic lane appears clear, the car’s sensors must operate at significantly longer ranges (and thus lower resolutions, with corresponding reduced certainty) to assure the car that this is the case.

Further, in the three-option cases, the differences between left and right swerves make no reference to which side the oncoming traffic lane is on. Instead, these options are differentiated by who will be affected by the crash. For Gogoll and Müller, the options are the car’s own occupants or the occupants of the passing car. For Lin, the choice is between a young girl and an old lady. For Woollard, it is a single pedestrian or a crowd of schoolchildren. It is far from clear that a self-driving car will actually be able to make distinctions of this type, and a later section of this paper establishes that the risks of designing it to try will outweigh any benefit. For this reason only two kinds of option merit consideration: the car either swerves, or continues straight, with each option involving danger to a different subset of participants in the scenario.

A significant disanalogy between all these cases and the classic formulations of the trolley problem is that, in classic formulations, one option is understood to be a course of *inaction*. That is, the agent refrains from interceding in the scenario in any way and thus only allows harm to occur rather than actively causing harm. However, while only Gogoll and Müller make explicit reference to braking in their scenario, it is reasonable to think that both kinds of option now under consideration assume that the car will do as much braking as is possible prior to collision.^2^ To put it another way, the sense in which the collisions are ‘inevitable’ in each case is that the obstacles described are within the car’s stopping distance. Perhaps the omission of braking from the scenarios as written is intended to preserve the analogy with the trolley problem; however, as will be shown below, the dynamics of braking significantly affect the outcome of any manoeuvre made during an accident.

One interpretation of the absence of braking in most of these cases might be that the brakes are presumed to have failed. This would maintain the analogy with traditional forms of the trolley problem, which specify that the vehicle involved is a runaway, out of control except for the pulling of the lever to change which track it follows. Embracing this reading would be a mistake, though, given the insistence of each author that cases such as these are inevitable and likely to be common enough to merit extensive consideration. Brake failures on modern vehicles are rare and seldom unpredictable. A self-driving car will have acutely sensitive monitors of the state of its brakes, and should certainly be programmed to insist on being serviced long before brake failure becomes likely—perhaps even to seek out its own servicing if its owner or operator is complacent.

Here, then, is a generic form of the self-driving car dilemma:

A properly-serviced self-driving car detects an imminent collision and must determine whether or not to swerve while braking; both braking in a straight line and swerving will result in collisions, but with different objects, neither of which is a roadgoing vehicle.

The solution to this dilemma, at least insofar as it concerns imminent technologies and their robustly foreseeable descendants (that is, those that can be foreseen without postulating radical transformations of what is currently available), is that the car should not swerve. Swerving is a drastic manoeuvre—the stipulation of any self-driving car trolley problem, that the objects in the car’s path are within its stopping distance and thus cannot be avoided by simply braking, entails that any steering the car does will be sharp relative to its speed, and thus at significant risk of loss of vehicle control. In any environment sufficiently crowded that all paths available to the car result in collisions, a loss of vehicle control is much more dangerous than a controlled stop.

Despite the extensive literature on the self-driving car trolley problem, very few writers even mention the possibility of a solution along these lines. Björn Meder and colleagues quote Andrew Chatham, an engineer at Google’s X project, saying in 2016:

It takes some of the intellectual intrigue out of the problem, but the answer is almost always ‘slam on the brakes’ (2019: 2)

And Matthias Rolf, Nigel Crook and Jochen Steil write that:

From a practical ethical perspective, simple breaking [sic] to minimize potential impact might be a better choice than attempting to make a rational choice of who to kill. (2018: 1)

But this suggestion has not been developed in any detail. Here, this solution will be put on firm theoretical footing. The argument proceeds as follows:

The next section establishes that, insofar as it can be applied to self-driving cars, the dilemma problem is one of risk management, and indeed of the management of *all* risks in the scenario, not merely those specified as relating to the posited outcomes. After that, the physics of vehicle dynamics are detailed and an explanation given of why swerving is inherently much riskier than braking in a straight line. For a self-driving car to be able to determine that the increased risk of swerving is (over)compensated for by the reduced risk associated with hitting a different object than the one immediately in front of it, it must have a great deal of detailed information about its environment. The following section makes the case that much of the necessary information is practically unavailable, either because of the level of detail required, the limitations of car-mounted sensors, or the privacy concerns bound up in connected and networked driving. This entails that it is extremely unlikely that a self-driving car could ever reasonably be required to swerve. Finally, a possible extension of this approach to scenarios involving multiple vehicles is suggested.

## Death and Certainty

Immediately after presenting their truck case, Nyholm and Smids quietly correct themselves:

[I]n order for the five passengers in the self-driving car to be saved, as they are *likely* to be if the head-on collision with the heavy truck is avoided, the car here needs to make a maneuver that will *most likely* kill one person. (2016: 1278, italics added)

The adjustment to probabilistic language is important for their overall project, which is to critique the presenting of these dilemma-style cases as analogous to the trolley problem. They present three arguments to this effect, and while it is the third that is most important for present purposes, it is worth briefly mentioning the other two.

Firstly, they point out, a critical difference between the trolley problem and the dilemma faced by the self-driving car’s controlling program is in the role of *urgency* in the situation. In the trolley problem—and, indeed, when a human driver is faced with the driving dilemma—a snap judgement is required about what should be done. The human being is required to decide in the moment, and legal processes afford some leniency due to the lack of time for consideration.^3^

In the trolley problem, it is assumed, the agent decides what *to* do at the same time they decide what *should* be done. These are treated as one and the same decision, even if it is hoped that an abstracted discussion of the scenario can influence and refine participants’ decisions.^4^ In the self-driving car case, however, the practical and normative decisions are less closely connected. The car must obviously ‘decide’ in the moment exactly what to do, but the decision about what it *should* do will be made in a lab or design meeting before the first production model rolls out of the factory.

Therefore, the grounds for leniency disappear; the designers of the self-driving car’s system have all the time in the world to decide what the car will value. Indeed, there is a plausible argument that releasing self-driving cars into the wild *without* exhaustive debate and robust conclusions on this issue is fundamentally irresponsible, especially as what the engineers settle on will apply across all cars made to their design. While time and urgency are still a factor in the generic dilemma outlined above, because no computer works instantaneously (for a detailed discussion, see Freichard 2014), they do not factor, or excuse imprecision, in moral assessments of what eventually occurs.

Secondly, Nyholm and Smids take up Wood’s (2011) critique of Derek Parfit, that the trolley problem as traditionally framed omits important contextual factors that, in an applied case, must be restored. If a bystander climbs a trackside fence to interfere with railway equipment, even if doing so for what they believe to be moral reasons, they risk interfering with safety systems of which they are ignorant. A signal operator employed by the rail company ought to behave in accordance with company safety policy; that policy ought to have a contingency which covers the situation and which may rely on the operator playing their role precisely while trusting others to play theirs.

One way in which this contextual critique applies to the self-driving car dilemma has already been described: the tunnel and tree cases fail to take into account that swerving towards the oncoming traffic lane is much less safe than swerving away from it. Another is that car safety features and general design anticipate certain kinds of crash being more common or otherwise more important to design for than others. As such, cars that behave in line with those expectations are likely to be safer than cars which radically diverge from them (though this does assume that car safety design stays more or less constant). In more detailed hypotheticals, there will be other factors that may seem relevant, though, as will be argued below, many of these factors should not ultimately be considered.

Both of these arguments sharpen the stakes around the self-driving car dilemma. They demand that the engineers and designers of self-driving car systems get more aspects of the ethical situation more securely ‘right’ than a human in an emergency-stop driving situation. However, since both arguments relate primarily to the obligations of the designers, it is not clear that they directly address the question of what the car should actually do.

It is Nyholm and Smids’ third argument that is central to the present case. They argue, as the adjustment quoted above suggests, that the attempt to *apply* the trolley problem brings in a new element not traditionally found in trolley problem scenarios: risk and uncertainty. In a traditional trolley problem, the possible outcomes are stipulated in the scenario and discussion is supposed to concern which of them it would be morally correct to bring about.

In the real world, though, and especially when driving, the outcome of a decision to follow one course of action over another can never be known with certainty. Most car accidents do not involve fatalities, and the range of factors that determine what casualties result from a collision is vast. Small changes to the angle or speed at which a car hits a pedestrian may make substantial differences to the resulting injuries. Different car behaviours may also provoke different responses from other agents in the environment, from leaping out of the way to panicked freezing in place.

The division just made between the obligations of the car’s designers and the car’s in-the-moment selecting of a course of action might be thought useful here. One could argue that the dilemma between certain outcomes is important precisely because it poses the question that the car’s designers must answer (i.e. ‘which of these outcomes is morally preferable?’) before they can develop a control program that best implements that answer.

This seems to be a concrete example of the spirit expressed by Derek Parfit:

In trying to answer such questions, it is best to proceed in two stages. We can first ask which acts would be wrong if we knew all of the morally relevant facts… After answering these questions, we can turn to questions about what we ought morally to do when we don’t know all of the relevant facts. These questions are quite different, since they are about how we ought to respond to risks, and to uncertainty. As in the case of non-moral decisions, though these questions have great practical importance, they are less fundamental. (2011: 162)

If Parfit is right, there are two kinds of moral question about a decision: one about which outcome is good and which bad, and the other, ‘less fundamental’, kind, which concern how a deciding agent should think about risk.

This separation of risk- and outcome-questions is untenable, however. Barbara Fried illustrates this with the example of a city deciding whether or not to construct a new sports stadium (2012: 515–516); large construction projects of this type carry innumerable risks of injury and even death, but with appropriate safety procedures, these risks are extremely small. Overall, building a stadium carries a very small risk of causing deaths. Meanwhile, not building a stadium carries no risk of causing death, though it carries significant risk of denying large numbers of sports fans their entertainment. How might the ‘less fundamental’ issue of risk be separated and removed from the moral question of whether the stadium should be built?

This attempted separation seems to reduce the decision to the question of whether one life, or a handful of lives, may be exchanged for some—perhaps even quite a lot—of sports entertainment. *But this is not the decision that city planners and the stadium’s backers must make*. It is very likely, in fact, that their decision is merely between the absence of a stadium and its presence. The difficult question, the one that the local government and the builders’ union are likely to disagree strongly with the backers over, is over what budget ought to be allocated to safety equipment and procedures in order to ensure that obligations to worker well-being are met. The risk component is not separable from the outcome component.

The question of ‘(how) should the stadium be built?’ is complex. It is not, as Parfit seems to think, purely technical, though it certainly has an a posteriori component (judgements about risk require deep understanding of how things stand and behave in the empirical world). As Sven Ove Hansson puts it:

There are no easy answers to questions such as what risks you are allowed to impose on one person in order to save another or what risks a person can be morally required to take in order to save a stranger. These are questions that present themselves to us as moral questions, not as issues for decision-theoretical reckoning to take place after the moral deliberations have been finished. (2012: 44)

This synthetic account of the scope of moral dilemma questions is daunting. However, it opens up one possibility that the posing of certain-outcome dilemmas excludes. This is the prospect that empirical factors brought in by the estimation of risk may collapse the dilemmaic character of the scenario. What looks like a dilemma when both outcomes are equally well-known but ambiguously valued may look much simpler when one option is a known and relatively controlled harm but the other is difficult to predict and bears an open risk of disaster.

## The Dynamics of Braking and Traction

The emphasis on driver reaction time in accident statistics and campaigns against speeding may give the impression that the emergency stop procedure—brake as hard as possible without skidding, in a straight line—is recommended because it simplifies the human element in driving safety. A human faced with an impending accident need not deliberate what to do; just brake. While this is doubtless an advantage of the procedure, though, it is not the fundamental reason why the emergency stop is preferred.

Instead, the justification is a matter of vehicle dynamics—the physics of motion, cars, and particularly tyre grip. For that reason, this explanation will be a bit on the technical side, but no sophisticated mathematical background should be necessary to understand it. Put in its simplest form, the problem is that swerving sufficiently to avoid an object that is within a car’s stopping distance is always a wildly risky manoeuvre compared to straight-line braking. Remember the problem clarified above:

A properly-serviced self-driving car detects an imminent collision and must determine whether or not to swerve while braking; both braking in a straight line and swerving will result in collisions, but with different objects, neither of which is a roadgoing vehicle.

The purpose of this paper is to solve a practical problem facing contemporary or near-contemporary technology. For this reason it is appropriate to limit the discussion to cars using rubber tires on conventional road surfaces. Given the extent of current road networks, the scale of infrastructure replacement needed to move away from this paradigm puts any circumstance that does so far in the future.

The first aspect of this case that bears highlighting is that the primary source of risk is the car’s momentum—the fact that it is a large mass in motion. Anything that comes into contact with it in its direction of travel will be subjected to a substantial force in the same direction. A pedestrian or cyclist who somehow manages to collide with a stationary car has a much lower chance of being injured than one who collides with a moving car (this is true even if the cyclist runs into the car at some speed, since their mass is much less than the car’s and thus their motion involves less energy).

This means that the best way to minimise the risk associated with a car’s motion is to reduce its speed, as much as possible in as short a time as possible. The oft-cited statistic that a pedestrian hit by a car doing 40 mph has an 80% chance of dying and a pedestrian hit by a car doing 30 mph has an 80% chance of living is a simplification of many different statistics, but points towards a robust statistical pattern. For example, Erik Rosén and Ulrich Sander conclude:

[A] strong dependence on impact speed is present, with the risk at 50 km/h [31 mph] being more than twice as high as the risk at 40 km/h [25 mph] and more than five times higher than the risk at 30 km/h [19 mph]… We also found that approximately 50% of all pedestrian fatalities had exposure to an impact speed between 50 km/h and 80 km/h [50 mph]. (2009: 540)

What needs to be examined, then, is whether turning during braking has a reasonable chance of reducing impact speed relative to straight-line braking. To understand why it does not, we need to bring in two principles from the physics of friction.

The first of these is the distinction between *static friction* and *kinetic friction*. A moderately heavy object on a moderately rough surface will resist a gentle push. If the force of the push is slowly increased, the object will eventually ‘come loose’ and start to slide. When it is sliding, the object will seem to offer less resistance to the push than it did while stationary. What has happened is that when the object started to move, it ceased to be subject to static friction with the surface and became subject to kinetic friction instead.

Static friction occurs between surfaces that are stationary relative to one another. Kinetic friction occurs when one surface slides over another. The reason that keeping the object in motion is easier than getting it moving to start with is that static friction generates greater force—that is, more resistance to motion—than kinetic friction (Gross et al. 2017: 195).

Perhaps counterintuitively, when a car is in normal motion and its wheels are turning, the friction between the tyre surface and the road surface (which is the limiting factor on all the important forces acting on the car) is static, not kinetic. The tyre contact patch is stationary relative to the patch of road surface it interacts with. The motion of the car is achieved because the turning of the wheel moves the contact patch along the surface of the tyre.

What all this means is that tyres grip better when rolling than sliding. This is a general rather than a universal truth—where both surfaces are very smooth there can be very little difference between kinetic and static friction—but it is reasonable to assume it applies consistently across ordinary tyres and road surfaces. It is for this reason that emergency stop guidance usually explicitly cautions against braking so hard that the wheels ‘lock’ (stop turning) and the car goes into a skid.

The maximum value of static friction—the resistance of an object in the moment before it starts to slide—is called the *limiting friction*. This can be thought of, at any given time, as a budget against which all forces applied to a tyre (by the acceleration, braking or turning of the car, for example) are charged. When the budget is exceeded—when the sum total of forces acting through the tyre is greater than the limiting friction—the tyre will cease to be governed by static friction and instead be governed by the weaker kinetic friction.

Critically, a car’s tyres are the *only* source of grip available for affecting its motion. To decelerate the car, a force must be applied in the opposite direction to the car’s travel, and it is the tyres’ contact patches with the road surface which allow the action of the car’s brake pads or discs to actually exert this force. Similarly, to turn the car away from its current direction of travel, a lateral force must be applied to the chassis, and again, it is only the contact of the tyres with the road surface that provides this force. When a car is turning *and* braking, the two forces are added together as far as the load on the tyre is concerned; both are charged against the same traction budget (Abe and Manning 2009: 24–25).

Thus, the more force one applies to changing the direction of the car’s travel, the less braking one can do without exceeding the limiting friction. If the car swerves, then, it is at much higher risk of skidding (that is, its tyres’ grip changing from static to kinetic friction), and even if a skid is avoided this can only be because it is doing less braking than it could be, which entails a higher-speed collision.

It is now possible to be more precise about the risks involved in swerving. There are two groups of risk at play here: the risks associated with skidding and the risks associated with higher-speed impacts.

On the first group, the motion of a car is more predictable when it is not skidding. This makes it easier for other road users to get out of the way. A car that brakes in a straight line rather than while turning is also at much lower risk of not merely losing traction but also going into a spin. Spinning is dangerous because a spinning car is less likely to hit whatever it hits squarely, head-on; car bonnets are designed both to minimise harm to pedestrians struck this way (by being slanted to lift and roll them out of the way^5^) and to protect the car’s occupants with a so-called ‘crumple zone’—D. C. Richards finds that:

It is much more likely that a driver will suffer a fatal injury if they are involved in a struck side impact. For a delta-v [speed of impact] of 30 mph, the risk of fatality in a frontal impact is 3% compared with 25% in a struck side impact. At 40 mph, the risk is 17% in a frontal impact compared with 85% in a side impact. (2010: 26)

The increased risks associated with higher-speed impacts have already been mentioned, but can now be given more detail. At the relatively low speeds (20–30 mph) of urban driving, where collision with pedestrians is most likely, small reductions in speed make large differences to fatality rate. At higher speeds, especially above 50 mph, this is less true; a pedestrian struck at 60 mph, for example, is not at much less risk of death than a pedestrian struck at 65 mph.^6^ However, because the momentum of the car increases with its velocity, greater force is needed to deflect it from its path at these speeds, which means that any swerving manoeuvre necessarily brings the car much closer to the limiting friction of its tyres. If an obstacle strays into a car’s path at such a distance that a straight-on collision will happen at over 50 mph even with optimal braking, any swerve sharp enough to avoid the obstacle will be absurdly risky.

So far, this discussion has focussed on cases in which the incident takes place on a straight road; that is, cases in which the car is not already turning when a response becomes necessary. Depending on circumstances, performing a conventional emergency stop in the middle of a corner may lead to the car travelling straight on into the oncoming traffic lane or spilling off the road altogether, either onto a pavement or into a crash barrier. All of these may be undesirable outcomes in their own right, so there is an important question of whether the car should turn while braking if doing so will allow it to stay in its own lane.

On this point the important thing to note is that (again for reasons of the vehicle dynamics outlined above) cornering should be done at reduced speed relative to straight-line driving, and the appropriate reduction in speed is proportional to how tight the corner is. The wide, sweeping turns of a motorway merit almost no reduction in speed at all, but the right-angle corners of a city block or the narrow twists of a country road must be taken much more slowly than their corresponding straights.

Further, the tightness of a corner determines how far the car can travel out of its lane before straying into oncoming traffic or off the road. Overall, then, where the car has less room to stray, it can also be expected to be travelling at lower speeds and thus have both a shorter overall stopping distance and more traction available to steer with while braking. This is not to say that emergencies while cornering are no problem at all, but it does entail that the problem they present is not as severe as might be expected.

To further mitigate the risks of this kind of situation, self-driving cars can be developed with a proactive approach to safety, such as is sketched by Johansson and Nilsson (2016). For example, where road lanes are narrower, or corners sharper, the car would proceed more slowly so as to minimise the risk of an emergency stop carrying it beyond the boundaries of its lane. This does not incur the problems of information-related risk which will be outlined in the next section because information about the boundaries of the car’s lane is fundamental to its ability to function at all; risk cannot be increased by the car collecting this information.

Cornering, then, does not present substantial problems for the strategy of preferring an emergency stop in all unavoidable-crash situations. This strategy has some other practically and morally relevant benefits. From an ethical-legal standpoint, the emergency stop is consistent with common principles of road law. This is an obvious practical advantage from the designers’ point of view, since it gives them a reasonable line of legal defence in the event of any such collision. But it also has a much broader practical significance, which is that it does not require widespread changes to other patterns of road-use behaviour. Asking all road users to be prepared for self-driving cars to behave radically differently to the human-driven cars they are used to introduces new difficulties and risks to driving in a great many other situations, and has the potential to create widespread turmoil.

Finally, it should be noted that self-driving cars are likely, and certain in the future, to be much better at performing an emergency stop than human drivers. They will have greater sensitivity to the limiting friction of their tyres and finer control over brake pressure. It is hoped that they will also be able to react more quickly, on average, than human drivers.

What this section has established is that the risks of any attempt by a self-driving car (or indeed a human-driven car) to swerve while braking greatly outweigh those of a straight-line emergency stop. The question of whether these risks can be outweighed by other considerations, such as the identities of the objects to be hit, is considered in the next section.

Here it is important to reemphasise the practical problem that this paper aims to solve, namely the question of how the car should (be programmed to) decide what to do. This decision cannot be made retroactively, by judging what actually did happen as a result of the decision, and any solution must be one that can be recommended as a general design, to be implemented identically across at least full production runs of individual vehicle models. The key considerations, then, concern what information can safely be made available to all self-driving cars and what risks its acquisition and analysis present.

## Higher-Information Hypotheticals and New Risks

The preceding section shows that the choice made by the car cannot be framed as it has generally been in the literature. The car does not face a decision between hitting an object in front of it and hitting an object off to one side. Instead, the decision is better described as being between a controlled manoeuvre—one which can be proven with generality to result in the lowest impact speed of any available option—and a wildly uncontrolled one. This is analogous to Fried’s refiguration of the stadium example discussed above.

The trolley problem, to do the ethical work it has generally been understood to do, requires the presentation of two options of roughly equal unattractiveness. In the case of the self-driving car, the raw physics of how vehicles interact with road surfaces means the two options, swerve or emergency stop, lack any such parity. Swerving is massively less attractive than the emergency stop, because of its unpredictability and the fact that it results in higher speed impacts.

To make an emergency stop sufficiently unattractive as to place it in the same ballpark as any swerve, then, there must be a radical difference in outcomes—that is, in the value to be placed on anything that the car might hit. And not only this, but, for the car’s designers to be justified in designing it to swerve in such cases, the car must be capable of discerning that such a value difference is present.

This presents obvious problems in the kind of scenarios that are typical in the literature. Woollard’s swerve case provides a particularly egregious example, in that part of its value difference consists in the fact that the pedestrians are drunk.^7^ There is no way for a self-driving car with anything resembling present technology to tell by sensors alone that a pedestrian is drunk; high blood-alcohol content has no unique externally-discernible symptoms (staggering, slurred speech, flushed cheeks and so on may all equally well be produced by any number of illnesses and disabilities, and indeed by involuntary intoxication).

In an enormous and widely-promoted study, Awad et al. (2018) found that people generally preferred that a car aim to hit a criminal rather than a control (a nondescript adult human), but slightly preferred for it to hit a control rather than an executive or athlete. If drunkenness is hard to discern externally, then one’s career or criminal record is surely much harder. Executives sometimes take off their suits; criminals do not generally wear black-and-white-striped jumpers and carry sacks marked with large dollar signs.

For a self-driving car to be able to identify the current physiological state, or biographical details, of pedestrians in its environment, it would have draw this information from a wide range of networked sources—government databases, perhaps identity cards or biomonitors carried by the pedestrians themselves. And the car could not simply wait until a crash was imminent to seek this information. Any trolley problem incident would, necessarily, appear and conclude in a matter of seconds, leaving the car without time to securely obtain answers from the various databases it would need to search.

The car, along with every other car using the same software, would have to be gathering any and all information deemed relevant in trolley scenarios from every other road user, at all times. There are obvious practical security risks to having this much data flying around, and it is also likely that pedestrians of greater means would find ways to ‘spoof’ the system by interfering with any data sources they carried. Even if these practicalities could be overcome, however, this scenario still involves a massive and systemic breach of civilian privacy rights (Holstein and Dodig-Crnkovic 2018: 4).

Should discrimination on any of these grounds be allowed? When the outcomes are treated as certain, and risk is removed altogether, these scenarios become referenda on the relative value of different kinds or categories of life or lifestyle. When lives hang in the balance—even only by stipulation—this is tantamount to making the right to life contingent on other values. Should a criminal really have *less of a right to life* than a law-abiding citizen? A drunk person than a sober? These are unpalatable questions, and quite rightly ruled out by a range of ethical codes from Kant’s categorical imperative to the IEEE code of ethics; most relevantly, the German Ethics Commission on Automated and Connected Driving (Federal Ministry of Transport and Digital Infrastructure 2017: 11) argues that this exclusion should be established as a consistent standard.^8^

Perhaps the most plausible scenarios in which a trolley problem-type dilemma might develop are those where the moral ambiguity is sustained by a difference in the number of people likely to be hit. It is tempting to say that there must be some number of people such that the risks of straight-line braking and crashing into them outweigh the risks of swerving towards a single person instead. Even here, though, there are a number of practical difficulties.

The first of these is that people are not transparent; sensors mounted on the car will necessarily be limited to relatively side-on perspectives on any crowd of people, and so will struggle to count how many people are behind those directly visible to it. Presented with three figures in the road ahead and two on the pavement, it may be literally impossible for the car to tell whether a third or fourth person is standing behind the two. Perhaps, again, the car could take advantage of nearby high-angle CCTV cameras to corroborate its count, but the same concerns apply here as above; the information would have to be more or less freely available to every passing self-driving car, with obvious knock-on privacy concerns.

Further, human beings are not all equally susceptible to harm. A car that emergency-stops into a crowd of sturdy, athletic young adults may do much less harm to them than one which swerves into a single elder at roadside (this is especially true given the risk that swerving might not result in the kind of head-on impact that car front ends are designed for). Again, these differences in vulnerability are not easy to identify by sensor alone; ordinary clothing may hide a great deal about variations in body types, and age manifests so differently in different people that reliable visual determinations of age are extremely difficult (Yoo et al. 2018: 808).

The behaviour of pedestrians in imminent-accident situations is also unpredictable, especially when presented with a wildly-swerving car. A car that identifies a large group of schoolchildren suddenly running out into the road in front of it, their teacher standing helplessly at roadside, is not in a position to reliably assume that the children will stay in their present locations if it swerves. They may instinctively try to leap back onto the pavement—into the path of the swerve—or, worse, scatter across the rest of the road, potentially into the path of other drivers as well. This is also true, of course, if the car does not swerve, but at least if the car does not swerve it is behaving in the way that is easiest for the children to predict and react to, and whatever impact occurs will be at the lowest possible speed.

Here it is again useful to emphasise the risk of startling other road users that attends on sudden swerving and lane changes. Any sudden lane change breaks the ordinary flow of road use and may distract or confuse other drivers into their own dangerous manoeuvres. And a car that mounts the pavement in an urban environment also risks that a pedestrian may step out of a shop, or out from behind a parked car or van, into its path—a busy street has an enormous variety of blind spots.

Swerving, then, generates a huge number of risks which are avoided or minimised by performing an emergency stop. Some of these relate specifically to the presence of humans in a scenario, but others would count against swerving even in the most straightforward hypothetical case. Consider a scenario in which an empty self-driving car is driving along a flat, straight road with flat verges, when the only person at roadside suddenly steps out in front of it. If the car does an emergency stop, it will hit the pedestrian, albeit at the minimum possible speed.^9^

What if the car swerves? To be certain of missing the pedestrian, the car must be sure that it can apply sufficient steering angle to actually avoid them. It must be sure that if it does so, the grip available from the road surface is consistent enough that it will not start to skid, which will likely lead to it side-swiping the pedestrian (assuming the pedestrian does not try to leap out of the way). If it swerves towards roadside, it must be sure that the surface of the verge has no hidden bumps that might unsettle the car into a skid, or ditches that might even pitch it into a roll.

A sufficiently advanced suite of RADAR and LIDAR sensors might be able to determine all of these factors (though some, such as estimating the consistency of grip available from the road surface, may depend on such miniscule variations as to put this beyond reach of anything it is practical to mount to a car-sized vehicle). It is unlikely that there are many stretches of road that pass all these tests, though, and even more unlikely still that such an idealised accident happens to occur on one.

Every complication that might be introduced into such a scenario adds further concerns that favour not swerving. If there is a raised kerb at roadside, its height and shape must be precisely measured before the risks of mounting it can be judged. If there is foliage on the verge, then the species and age of the trees and bushes, as well as the quality of their planting and the condition of the soil, may make significant differences to their stopping power. Any solid object that obscures a sufficiently large part of the car’s view might hide a pedestrian, who might be injured if the car knocks the object over.

Some of these concerns, such as kerb heights, might eventually be solved by data collection. The privacy concerns that count against giving self-driving cars access to the criminal records database do not trouble objective environmental measurements to the same degree. However, collecting all this information is a massive undertaking, and so is the task of keeping it up-to-date—road surface data, for example, would have to be updated every time a pothole formed or was fixed. Even if it could be done efficiently, this mapping still lies in the relatively distant future.

All of the above counts against any car swerving, not just self-driving cars. Human drivers should not swerve either, and indeed, this policy is well-supported by road law in many major jurisdictions. Perhaps for this reason, none of the classic examples in the trolley problem literature pose the agent as the driver of a roadgoing car. There is no good reason to think that self-driving cars should ever find it preferable to swerve rather than simply performing their best emergency stop in any imminent-accident case where their own motion is the primary source of risk of harm. The question of whether this solution extends to multiple-vehicle cases is considered in the next section.

## Multiple-Vehicle Cases

Nyholm and Smids’ truck case is perhaps the most difficult problem of this kind. A head-on collision with a much larger vehicle moving under its own power is one of the most dangerous things that can happen to an ordinary car. Braking, even to a halt, will do relatively little to neutralise the force of the collision. Should the car then swerve?

This question is, from a risk point of view, absurdly complex. Many of the variables discussed at the end of the last section are suddenly unavoidably relevant; the distance to the truck, the extent to which it is out of its lane and continuing to stray, the width of the road, the distance to the pedestrian at roadside, the height of the pavement kerb, the presence of other obstacles alongside the road, the relative positions of other vehicles, and so on.

If there is a large enough distance between the car and the truck, and the kerb is low, it may indeed be safer for the car and its occupants in this circumstance to mount the kerb and then do an emergency stop. On the other hand, if the truck is truly out of control and its direction is unpredictable, and the street is narrow or crowded with other cars, then there may be no safe place for the car to swerve *to*.

In any permutation, however, sharp and controlled braking is unlikely to *increase* the risk to the car’s occupants and to bystanders. A slower-moving car will turn more sharply if a manoeuvre is necessary, and will be easier to put into reverse if that is likely to help. Braking will also increase the distance the truck must travel before hitting the car, giving its driver more room to correct. As in the single-vehicle case, the only risk that is directly increased by sharp braking is of being run into from behind by a tailgater.

This is a serious risk, although it is one that is already accounted for in much of modern car design. One of the reasons that cars have generally stuck with the template of a passenger compartment in the middle of the chassis with the engine ahead and a large storage compartment behind is that the engine and trunk sections of the chassis offer substantial protections to passengers. Nose-to-tail collisions are common, but relatively undramatic except where the impact speed is extremely high.

Further, nose-to-tail collisions, especially those in which the lead vehicle brakes sharply (which is to say, those in which the tailing vehicle is not maintaining safe distance) will generally be of lower impact speed than any other common kind of road accident, because the two vehicles by stipulation must be travelling at roughly the same speed when the braking event begins. Where that speed is high, both vehicles may behave chaotically after impact as either or both may be pitched into a spin or even a roll, but the actual force of the impact itself will be relatively small.

So, again, the risks turn out to be smaller than expected. Further, the car’s AI cannot be found at fault for an emergency stop in which a car behind it runs into its tail; by general legal principle, this will be the fault of the trailing car’s driver (or software) for not maintaining safe distance. Still, there are nevertheless circumstances in which it is *safer* for a human driver to consider the position of a trailing vehicle in deciding how to drive. Injuries such as whiplash are common in passengers of vehicles struck from behind, and can be severe (Krafft et al. 2004).

The question facing the designers of self-driving car systems is this: should a self-driving car, as a general principle of design, ever increase the risk it imposes on things ahead of it (by not braking as hard as possible) in order to reduce the risk imposed on it, its occupants and other road users by a trailing vehicle?

Say that the obstacle that has appeared in front of the car suddenly is a tarpaulin that has just blown loose from the back of a vehicle in the adjacent lane, and that the car is being tailgated by a large truck. Judging the situation by outcomes, the best option for the car is clearly not to break so sharply that the truck runs into it (the car should nevertheless brake as much as it can because the tarpaulin will block its view of the road ahead at least momentarily). However, all the concerns raised in the “Higher-Information Hypotheticals and New Risks” section apply equally here; how much discretionary judgement should the car be afforded about the objects in its environment? What objects should be deemed ‘worth the risk’ of running into to avoid an impact from behind?

In nose-to-tail multiple-vehicle collision scenarios, there are not quite the easy answers that were provided above for single-vehicle cases. It is more likely in the multiple-vehicle case that an emergency stop by the lead vehicle will lead to greater harm than a more gentle braking event that does allow a collision with an object in the road ahead (even though the moral fault for that harm will not lie with the car performing the emergency stop). Nevertheless, given the requirement that any self-driving car control system must be mass-produced and mass-implemented, the risks of a general policy that does not always err on the side of caution vis-à-vis objects ahead of the car are substantial, especially if the car’s information environment is to be rich enough for it to make reliable value-judgements about those objects.

## Conclusion

In situations where a self-driving car must choose between straight-line braking into an unavoidable collision and swerving into an unavoidable collision, where there are no other cars involved, the car should always prefer the straight-line option. Additional information about the objects to be collided with is irrelevant, since there is no way for the car to gather that information without making the risks of the situation worse. Finally, some incomplete justification has been offered for the claim that even in the much more complex situations produced by involving more vehicles, an emergency stop policy is at least good enough to be worth considering.

## Footnotes

1 Nyholm and Smids collect some representative examples (2016: 1276, especially footnote 1).

2 While the absence of a bystander option strains the analogy between the self-driving car hypothetical and the classic trolley problem, the decision faced by the car in scenarios of this kind carries obvious ethical weight and thus still does at least some of the work it is taken to do in the literature.

3 It should be noted here, though, that in the UK the law is extremely clear about what a human driver in a pending-collision situation should do: rule 118 of the Highway Code reads “In an emergency[:] Brake immediately. Try to avoid braking so harshly that you lock your wheels. Locked wheels can lead to loss of control.” (DfT 2018) This was the guiding principle behind much of this paper’s argument.

4 As Bostyn et al. (2018) show, the relationship between people’s responses to ethical dilemmas considered as hypotheticals and their behaviour when such a scenario is brought to life in an experimental condition is complex, and abstracted deliberations may not be much guide to people’s real-life ethical behaviours.

5 It should be noted here that the rising trend in favour of higher- and blunter-fronted SUVs over traditional cars has been linked to a shocking 46% increase in pedestrian fatalities in America since 2009 (Lawrence et al. 2018). This serves as a good illustration of how much difference the design of a car’s front end can make.

6 The author would like to thank an anonymous reviewer for this clarification.

7 An anonymous reviewer suggests that the drunkenness may be mentioned only as a heuristic, to explain how it is that three pedestrians come to be in the car’s path and within its stopping distance. This would be inconsistent with other trolley problem-style hypotheticals—including, for example, the cited cases from Lin (2016) and Gogoll and Müller (2017) in which all details mentioned are generally taken to be morally salient. Indeed, the strong moralising associations of drunkenness make it a poor heuristic; Woollard could equally well have specified that the three pedestrians had just leapt out of the way of some dramatic event on the pavement, that they had emerged from a blind corner, or that they had been lost in conversation and not looked carefully where they were going, to the same heuristic effect with no such moral implication.

8 An anonymous reviewer points out that discrimination on the basis of age, as for example in Lin's scenario, is allowed when deciding how to distribute donated organs. Obviously it is regrettable when such a decision is necessary; given that good grounds have already been established for thinking that the self-driving car can determine how to act without such an assessment, at very least there is reason to think that age-based decisions should be avoided here. A related concern, that military drafts often discriminate by age, can probably be attributed more to worries about the physical capability of draftees than morality, and rendering it *as* a moral concern would beg the question of whether military drafts can ever be morally justified, which is too large to examine in the present study.

9 It's not clear that a scenario like this has the kind of features which make the trolley problem and its application to self-driving cars interesting from the perspective of moral theorising, but it is nevertheless worth considering this case as an illustration of how demanding the swerve is.
